# Production of porous Calcium Phosphate (CaP) ceramics with aligned pores using ceramic/camphene-based co-extrusion

**DOI:** 10.1186/s40824-015-0037-z

**Published:** 2015-07-03

**Authors:** Won-Young Choi, Hyoun-Ee Kim, Young-Wook Moon, Kwan-Ha Shin, Young-Hag Koh

**Affiliations:** Department of Materials Science and Engineering, Seoul National University, Seoul, 151-742 Korea; School of Biomedical Engineering, Korea University, Seoul, 136-703 Korea

**Keywords:** Calcium phosphate, Porous materials, Pores, Scaffold, Bone regeneration

## Abstract

**Background:**

Calcium phosphate (CaP) ceramics are one of the most valuable biomaterials for uses as the bone scaffold owing to their outstanding biocompatability, bioactivity, and biodegradation nature. In particular, these materials with an open porous structure can stimulate bone ingrowth into their 3-dimensionally interconnected pores. However, the creation of pores in bulk materials would inevitably cause a severe reduction in mechanical properties. Thus, it is a challenge to explore new ways of improving the mechanical properties of porous CaP scaffolds without scarifying their high porosity.

**Results:**

Porous CaP ceramic scaffolds with aligned pores were successfully produced using ceramic/camphene-based co-extrusion. This aligned porous structure allowed for the achievement of high compressive strength when tested parallel to the direction of aligned pores. In addition, the overall porosity and mechanical properties of the aligned porous CaP ceramic scaffolds could be tailored simply by adjusting the initial CaP content in the CaP/camphene slurry. The porous CaP scaffolds showed excellent *in vitro* biocompatibility, suggesting their potential as the bone scaffold.

**Conclusions:**

Aligned porous CaP ceramic scaffolds with considerably enhanced mechanical properties and tailorable porosity would find very useful applications as the bone scaffold.

## Background

Porous bioceramics with an open porous structure have been widely examined as the scaffold for bone regeneration, since they can provide 3- dimensionally interconnected pores and biocompatible frameworks for cell attachment, proliferation and differentiation, as well as new bone formation *in vivo* [1, 2]. In particular, calcium phosphate (CaP) ceramics have gained much attention as a scaffold material on account of their similarity with natural bone in terms of chemical compositions and crystalline structure [3, 4]. These biomimetic physical and chemical characteristics allow porous CaP scaffolds to provide strong direct bond with the host bone *in vivo* as well as reasonable biodegradation nature when used as a bone scaffold [5].

Thus far, a variety of manufacturing techniques have been developed for the production of porous ceramic scaffolds [6], which include sponge replication [7], direct foaming techniques [8–12], vacuum-assisted foaming of a ceramic suspension (VFC) [13, 14], and freeze casting [15–17]. Fundamentally, the mechanical properties and biological function of porous ceramic scaffolds are strongly affected by their porous structure, such as porosity, pore geometry, pore size, and pore connectivity, as well as pore orientation [2, 18]. In general, high porosity is beneficial to bone ingrowth into 3-dimensionally interconnected pores but inevitably causes a severe reduction in mechanical strength [2, 19]. Thus, considerable effort has been made to improve the mechanical properties of porous ceramic scaffolds without sacrificing their high porosity. Unidirectional freeze casting is one of the most promising approaches for this goal, which can create aligned pores by inducing the preferential growth of ice dendrites along the direction of freezing [20–22]. The degree of pore alignment can be significantly enhanced by adopting polymeric additives [23–26], double-side cooling [27], and electric field [28, 29]. On the other hand, the use of camphene as a novel freezing vehicle allows for the production of porous ceramics with aligned pores even at room temperatures, which would provide more flexibility in manufacturing process [30–32]. Porous ceramic scaffolds with an aligned porous structure produced using these technique can have much higher compressive strengths than those with a random porous structure [33].

In this study, we produced porous CaP ceramics with aligned pores using ceramic/camphene-based co-extrusion, the basic concept of which was recently developed by our group [34, 35], and characterized their porous structure, mechanical properties, and *in vitro* biocompatibility for assessing their potential as a bone scaffold. The porous structure of porous CaP ceramic scaffolds (*e.g*. porosity, pore size, pore alignment, and pore connectivity) was characterized by field emission scanning electron microscopy (FE-SEM). The crystalline structure and phases were examined by X-ray diffraction (XRD). The compressive strength of the porous CaP scaffolds with aligned pores was measured to determine their structural integrity, while the *in vitro* biocompatibility of the scaffold was evaluated by *in vitro* cell tests using a pre-osteoblast cell line.

## Methods

### Materials

Commercial CaP powder (NT-BCP, OssGen Co., Korea) with a mean particle size of 0.5 μm was used as the starting material, while camphene (C_10_H_16_, Alfa Aesar/Avocado Organics, Ward Hill, MA, USA) were used as the freezing vehicle. CaP powder was comprised of hydroxyapatite (HA) and β-tricalcium phosphate (β-TCP) with a weight ratio of 60:40 (manufacturer’s specification).

### Preparation of CaP/camphene slurries and extrusion process

**Fig. 1:**
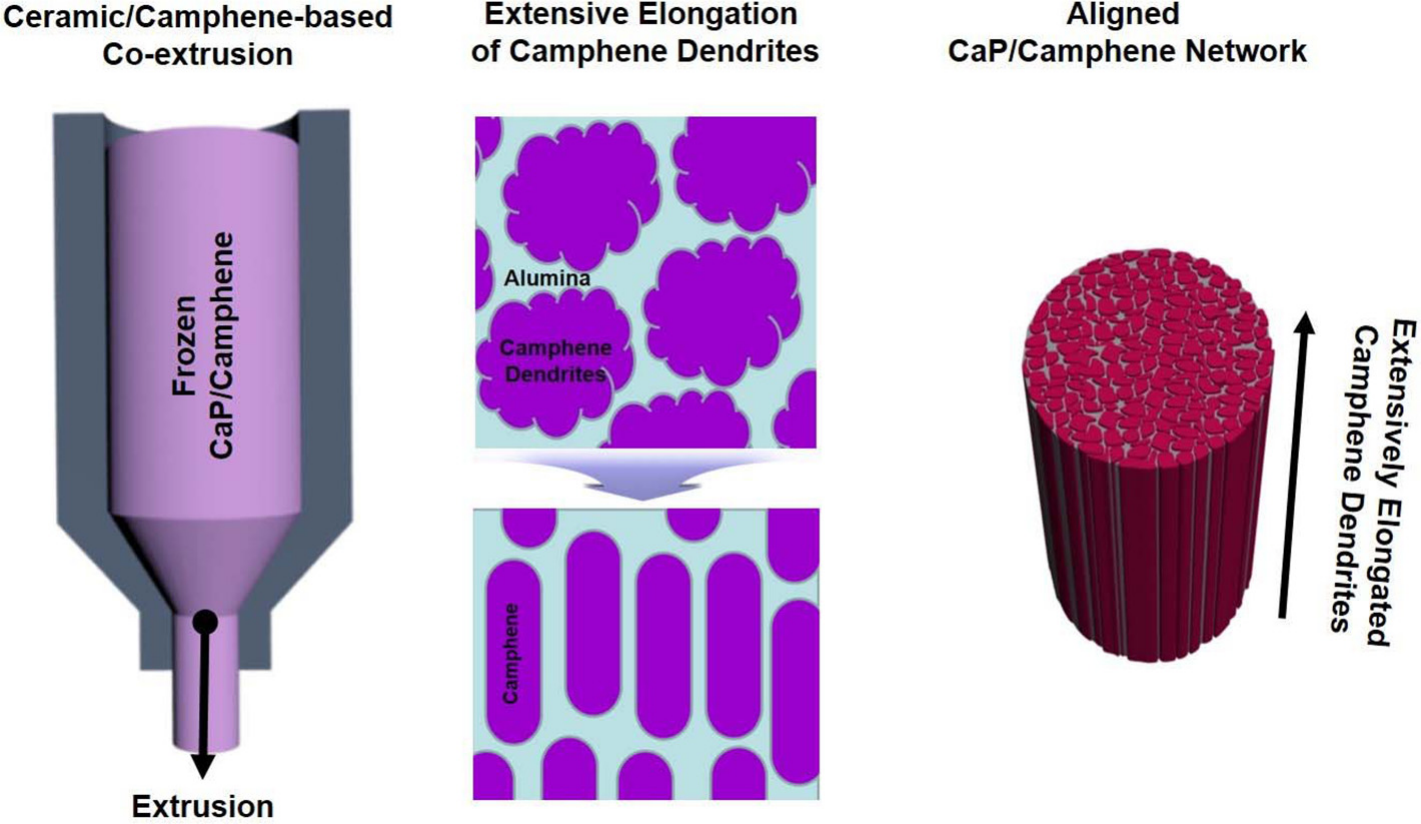
Schematic diagram showing the production of aligned porous CaP ceramic scaffolds using camphene-based co-extrusion, where camphene dendrites can be extensively elongated by extrusion process

CaP/camphene slurries with various CaP contents (15 vol%, 20 vol%, and 25 vol%) were prepared by mixing the CaP powder and molten camphene with 3 wt% of an oligomeric polyester dispersant (Hypermer KD-4, UniQema, Everburg, Belgium) using ball-milling at 60 °C for 24 h. The prepared CaP/camphene slurries were poured into metallic molds with a diameter of 20 mm and then frozen at 3 °C. The frozen CaP/camphene green bodies were then extruded through a reduction die with a diameter of 3 mm at room-temperature, which would cause the extensive elongation of camphene dendrites formed in the frozen CaP/camphene body (c.f. Fig. 1).

### Post treatment and sintering

To increase the pore size of porous CaP ceramics, the extruded CaP/camphene green samples were treated in an oven at 33 °C for 3 h in an oven for inducing overgrowth of the camphene dendrites [35]. After which, the green bodies were freeze-dried to remove the camphene dendrites, followed by sintering at 1250 °C for 3 h to densify the CaP frameworks.

### Characterization of porous structure and crystalline phases

The overall porosity of aligned porous CaP ceramics produced with various CaP contents (15 vol %, 20 vol %, and 25 vol%) was calculated from their dimensions and weight. The pore structure of the porous scaffolds was characterized by field emission scanning electron microscopy (FE-SEM; JSM-6701 F; JEOL Techniques, Tokyo, Japan). The pores sizes of the porous scaffolds before and after treatment at 33 °C for 3 h were also measured from their FE-SEM images. The crystalline structures and phases of the samples were characterized by X-ray diffraction (XRD, M18XHF-SRA, MacScience Co., Yokohama, Japan).

### Measurement of compressive strength

Compressive strength tests were carried out to evaluate the mechanical properties of aligned porous CaP ceramics produced with various CaP contents (15 vol%, 20 vol%, and 25 vol%). The samples before and after treatment at 33 °C for 3 h were tested. Samples (~2.9 mm in diameter and ~ 6 mm in height) were uniaxially compressed at a constant crosshead speed of 1 mm/min using a screw driven load frame (OTU-05D; Oriental TM Corp., Korea). The mean value and standard deviation were obtained from five samples.

### Assessment of in vitro biocompatibility

The *in vitro* biocompatibility of aligned porous CaP ceramics produced with various CaP contents (15 vol%, 20 vol%, and 25 vol%) was evaluated using a pre-osteoblast cell line (MC3T3-E1; ATCC, CRL-2593, Rockville, MD, USA). The MC3T3-E1 cells were plated at a density of 5 × 10^4^ cells/mL and cultured in a humidified incubator in an atmosphere containing 5 % CO_2_ at 37 °C. Minimum essential medium (α-MEM: Welgene Co., Ltd., Seoul, Korea) supplemented with 10 % fetal bovine serum (FBS), 1 % penicillin-streptomycin, 10 mM β-glycerophosphate (Sigma) and 10 μg mL^−1^ ascorbic acid was used as the culturing medium. The morphologies of the attached cells on the porous CaP ceramics after 24 h of culturing were examined by FE-SEM. In addition, cell viability after 3 days of culturing was examined using a MTS (methoxyphenyl tetrazolium salt) assay with 3-(4,5-dimethylthiazol-2-yl)-5-(3-carboxymethoxyphenyl)-2-(4-sulfophenyl)-2H-tetrazolium (MTS, Promega, Madison, WI, USA) for mitochondrial reduction [36].

### Statistical analysis

All quantitative data were expressed in terms of mean ± standard deviation (SD) values. One-way ANOVA followed by Bonferroni’s post hoc comparison tests were performed in all statistical analyses and *p* < 0.05 was considered significant.

## Results and discussion

### Change in pore geometry by extrusion

**Fig. 2:**
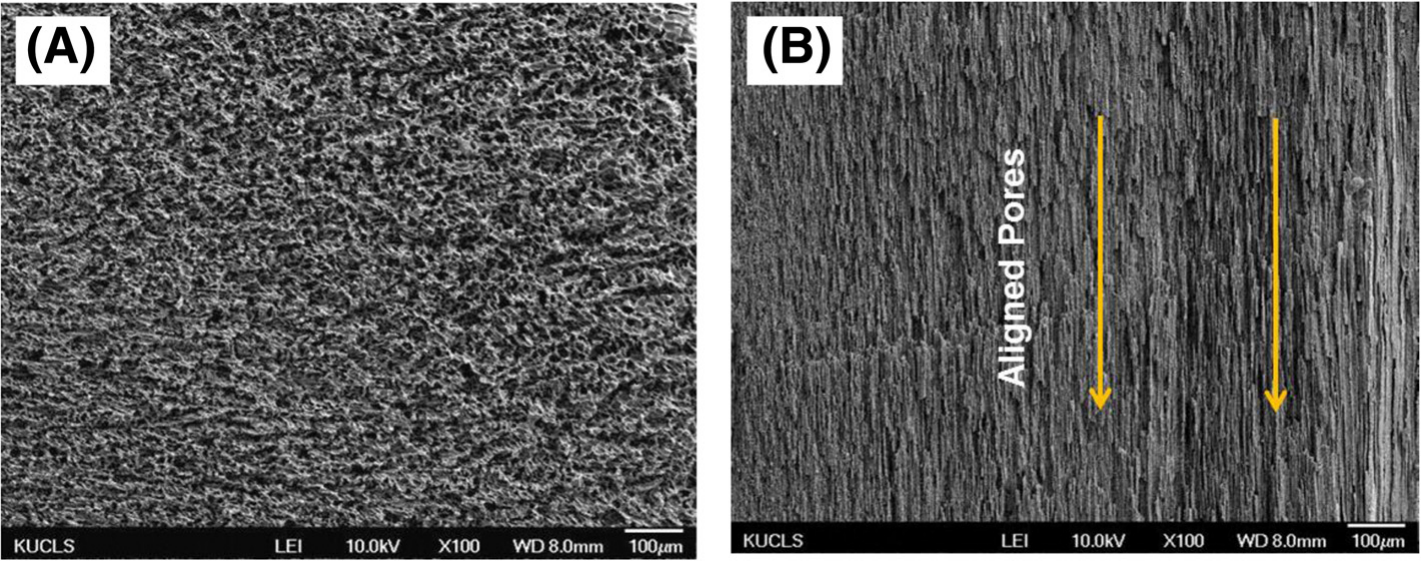
Representative FE-SEM images of porous CaP scaffolds produced (**a**) without and (**b**) with extrusion process

Porous CaP ceramics with an aligned porous structure were successfully produced by ceramic/camphene-based co-extrusion. In this technique, camphene dendrites formed in a frozen CaP/camphene body can be extensively elongated by extrusion (c.f., Fig. 1), accordingly, allowing for the creation of aligned pores after the removal of the camphene dendrites via freeze drying [34]. Figure 2 (a) and (b) show representative FE-SEM images of porous CaP ceramics produced without and with ceramic/camphene-based co-extrusion, respectively. The sample produced without extrusion process showed a number of pores with a random orientation, which is a general characteristic of camphene-based freeze casting [30, 34] (Fig. 2 (a)). On the other hand, the sample produced with ceramic/camphene-based co-extrusion revealed a highly aligned porous structure, owing to the construction of extensively elongated camphene dendrites by extrusion process [34, 35] (Fig. 2 (b)).

### Characterization of aligned porous structure

**Fig. 3:**
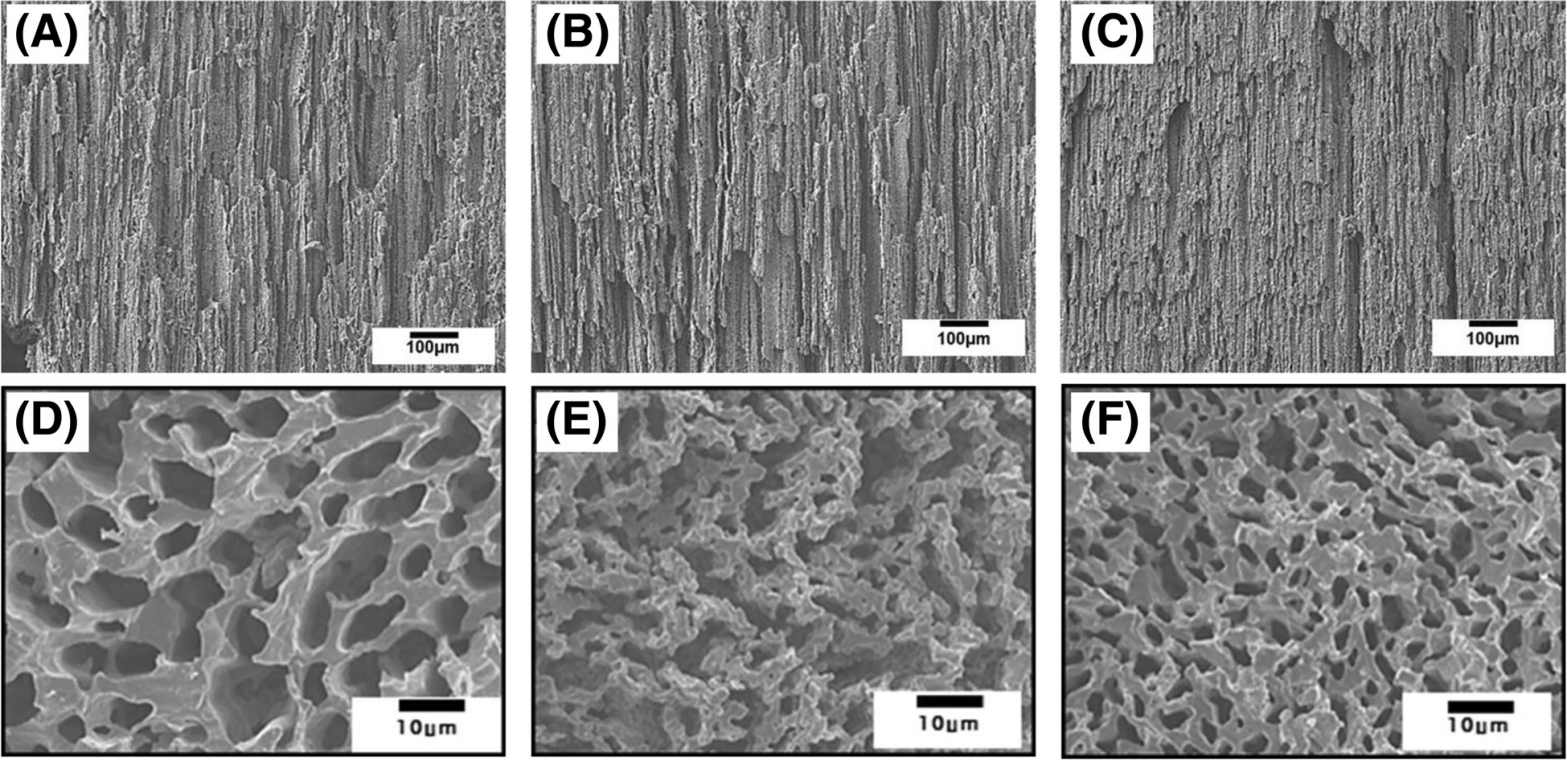
Representative FE-SEM images of aligned porous CaP scaffolds produced with various CaP contents (15 vol% : (**a**),(**d**), 20 vol%: (**b**),(**e**), and 25 vol% : (**c**),(**f**)), showing their porous structures developed parallel to ((**a**),(**b**),(**c**)) and normal to ((**d**),(**e**),(**f**)) the direction of extrusion

The pore structure of the aligned porous CaP scaffolds produced with various CaP contents (15 vol%, 20 vol%, and 25 vol%) was characterized by FE-SEM, as shown in Fig. 3 (a)-(f). Regardless of initial CaP contents, all of the samples showed a highly aligned porous structure, which was developed parallel to the direction of extrusion (Fig. 3 (a)-(c)). In addition, the cross-sectional views of the samples showed the creation of a open porous structure (Fig. 3 (d)-(f)). However, pores became narrower with increasing initial CaP content. This finding suggests that highly aligned pores could be successfully constructed simply by extruding a frozen CaP/camphene body.

### Effect of post treatment on pore size

**Fig. 4:**
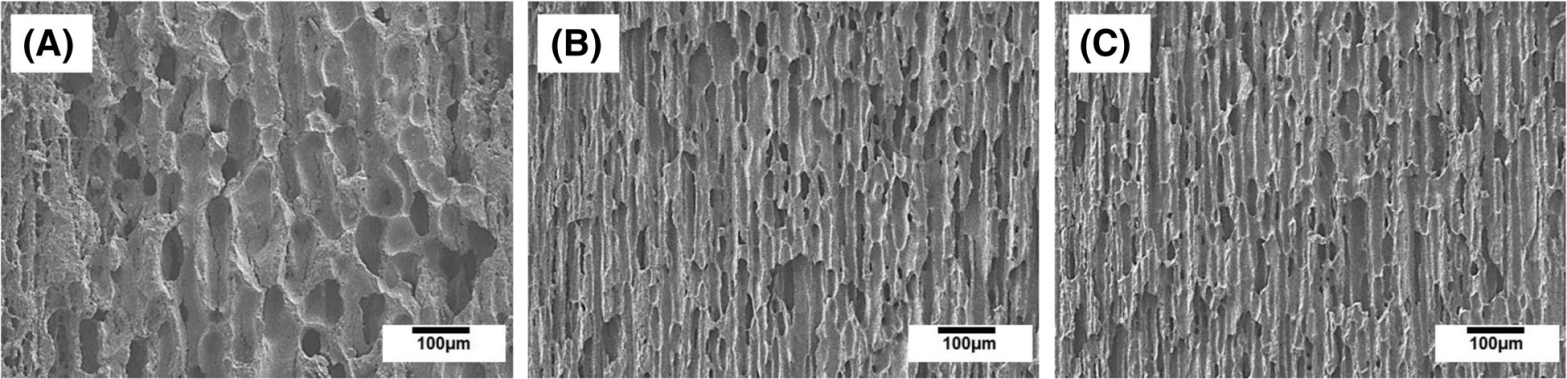
Representative FE-SEM images of aligned porous CaP scaffolds produced with heat-treatment at 33 °C for 3 h (CaP contents: (**a**) 15 vol%, (**b**) 20 vol%, and (**c**) 25 vol%)

The effect of heat-treatment at 33 °C for 3 h on the development of aligned pores was examined by FE-SEM. Regardless of initial CaP contents, all of the samples showed relatively very large pores compared to those produced without heat-treatment, while a highly aligned porous structure was well preserved, as shown in Fig. 4 (a)-(c). This finding suggests that the pore size of the aligned porous CaP scaffolds can be remarkably increased through simple heat-treatment at temperatures that is close to the melting point of the frozen CaP/camphene body [35].

**Fig 5:**
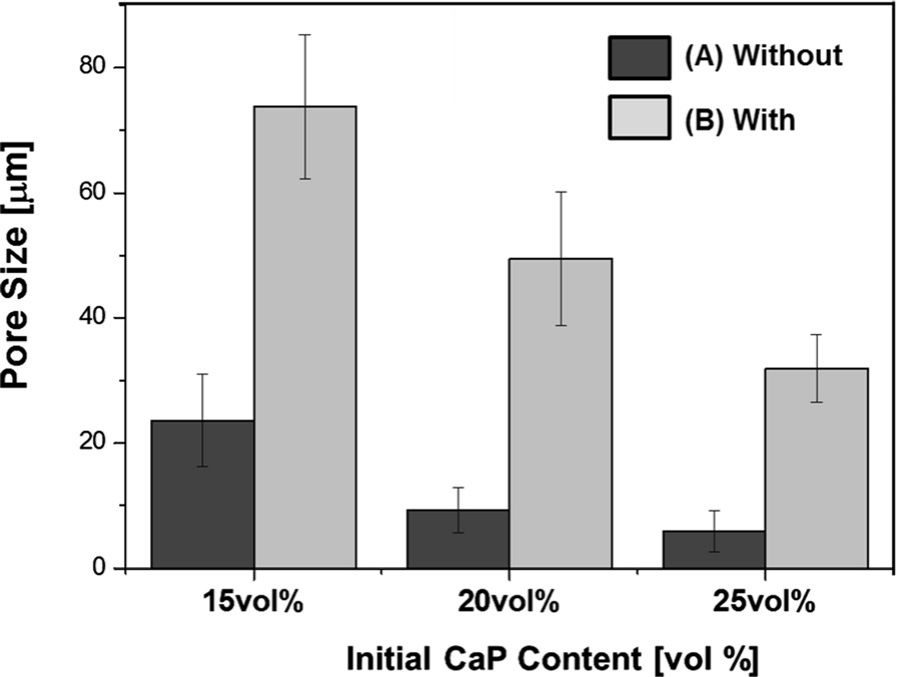
Pore sizes of aligned porous CaP scaffolds produced without and with heat-treatment at 33 °C for 3 h (CaP contents: 15 vol%, 20 vol%, and 25 vol%). The pore sizes between the samples are significantly different (*p* < 0.05)

The pore sizes of the aligned porous CaP scaffolds produced without and with heat-treatment at 33 °C for 3 h were calculated from their FE-SEM images, as shown in Fig. 5 (a) and (b). Both samples showed that the pore size decreased with increasing initial CaP content as is often the case with freeze casting [34, 35]. However, the pore size increased remarkably after heat-treatment at 33 °C for 3 h for all the samples. It should be noted that pores obtained using the present technique are 3-dimensionally interconnected, which would provide a favorable environment for bone ingrowth into pores [2].

### Compressive strengths of aligned porous CaP scaffolds

**Fig. 6:**
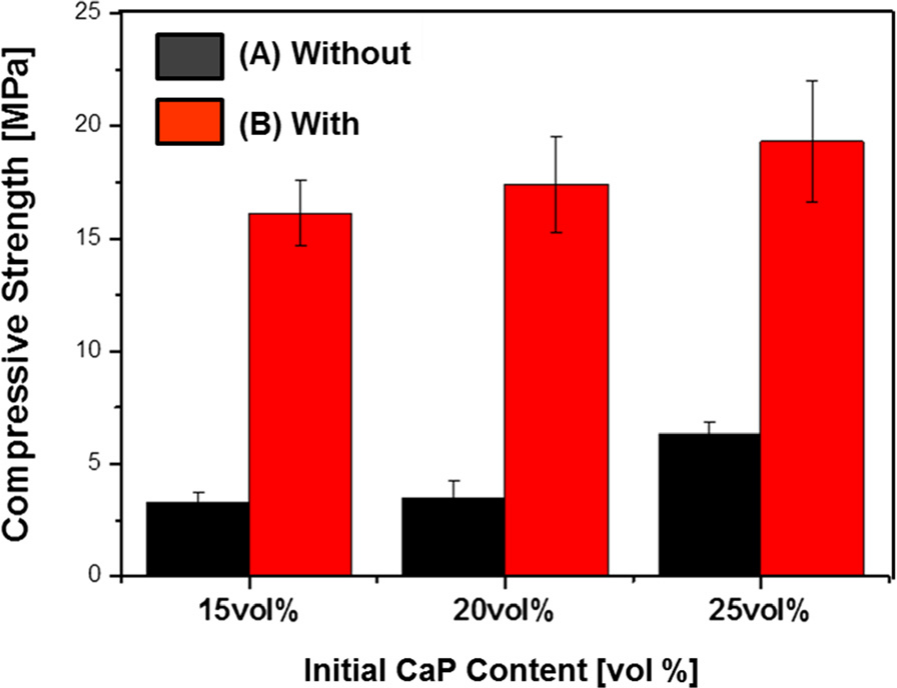
Compressive strengths of aligned porous CaP scaffolds produced without and with heat-treatment at 33 °C for 3 h (CaP contents: 15 vol%, 20 vol%, and 25 vol%). The compressive strengths between the samples are significantly different (*p* < 0.05)

Compressive strength tests were conducted for the aligned porous CaP scaffolds produced with various CaP contents (15 vol%, 20 vol%, and 25 vol%), in order to evaluate their mechanical function as a bone scaffold. The compressive strength increased from 3.3 ± 0.42 MPa to 6.3 ± 0.57 MPa with increasing initial CaP content from 15 vol% 25 vol%, as shown in Fig. 6 (a). This improvement was mainly attributed to a decrease in the overall porosity of the aligned porous CaP scaffolds, as summarized in Table 1. Furthermore, the compressive strength remarkably improved after heat-treatment at 33 °C for 3 h (Fig. 6 (b)), which was attributed to the achievement of well densified CaP frameworks. The aligned porous CaP scaffold produced with an initial CaP content of 25 vo% had a high compressive strength of 19.3 ± 2.7 MPa. It should be noted that porous CaP scaffolds with aligned pores can have much higher compressive strength than those with a random porous structure [34, 35].

**Table 1:** Overall porosity of aligned porous CaP scaffolds produced with various CaP contents (15 vol%, 20 vol%, and 25 vol%)

Initial Cap Content [vol%]	15	20	25
Overall Porosity [vol%]	71 ± 4.6	63 ± 3.8	55 ± 6.6

### *In vitro* biocompatibility

**Fig. 7:**
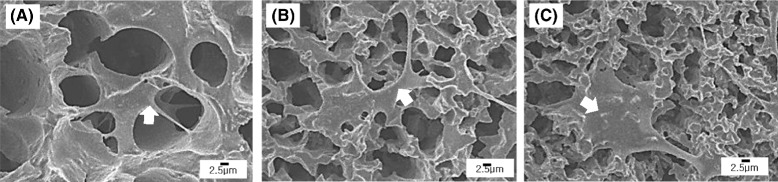
Representative FE-SEM images of the MC3T3-E1 cells on aligned porous CaP scaffolds produced with various CaP contents: (**a**) 15 vol%, (**b**) 20 vol%, and (**c**) 25 vol%. Arrows indicate cells attached on the samples

To evaluate the potential of the porous CaP ceramics with aligned pores for bone tissue regeneration, their *in vitro* biocompatibility was evaluated using a pre-osteoblast cell line (MC3T3-E1). Basically, all of the samples showed that a number of the cells, indicted by the arrows, adhered to and spread actively on the surfaces of the CaP frameworks after 24 h of culturing, as shown in Fig. 7 (a)-(c). This was attributed to excellent osteoblast activity owing to the co-existence of HA and β-TCP phases [5]. This finding suggests that aligned porous CaP scaffolds would provide a favorable environment for bone ingrowth.

**Fig. 8:**
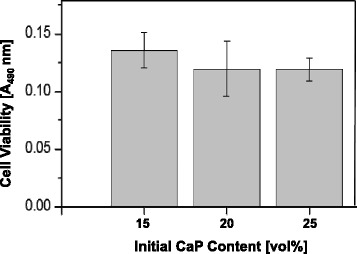
Cell viability on aligned porous CaP scaffolds produced with various CaP contents (15 vol%, 20 vol%, and 25 vol%). The values between the samples are not significantly different (*p* > 0.05)

The degrees of cell proliferation on the aligned porous CaP scaffolds produced with various CaP contents (15 vol%, 20 vol%, and 25 vol%) were examined after 3 days of culturing using an MTS assay, as shown in Fig. 8. All of the samples showed similar values without significant difference (*p* > 0.05). This finding suggests that the aligned porous CaP scaffolds produced ceramic/camphene-based extrusion can have excellent biocompatibility *in vitro*. However, it should be noted that further and more detailed *in vitro* and *in vivo* experiments should be conducted for clarifying the utility of porous CaP scaffolds with an aligned porous structure as a bone scaffold. In addition, the porosity and mechanical properties should be properly balanced to provide both structural integrity and fast bone ingrowth when used as the bone scaffold.

## Conclusions

Porous CaP ceramic scaffolds with an aligned porous structure were sucesfully produced by ceramic/camphene-based co-extrusion. Highly aligned pores could be created by removing extensively elongated camphene dendrites formed via extrusion process. In addition, the pore size could be significantly increased through simple heat-treatment at 33 °C, which is close to the melting point of the CaP/camphene slurry. Interestingly, this heat-treatment led to a considerable improvement in compressive strength. The aligned porous CaP scaffolds showed excellent *in vitro* biocompatibility. All of these findings suggest that porous CaP scaffolds with a unique aligned porous structure, coupled with tailorable porosity, high mechanical properties, and excellent biocompatibility, would find very useful applications as a bone scaffold.
